# Impact of AI-Assisted Diagnosis on American Patients’ Trust in and Intention to Seek Help From Health Care Professionals: Randomized, Web-Based Survey Experiment

**DOI:** 10.2196/66083

**Published:** 2025-06-18

**Authors:** Catherine Chen, Zhihan Cui

**Affiliations:** 1 Manship School of Mass Communication, Louisiana State University, Baton Rouge, LA, United States; 2 Department of Political Science, Louisiana State University, Baton Rouge, LA, United States; 3 Institute of Global Health and Development, Peking University, 5 Yiheyuan Road, Haidian District, Beijing, 100871, China, 86 15524772497; 4 Anderson School of Management, University of California, Los Angeles, Los Angeles, CA, United States

**Keywords:** AI-assisted diagnosis, patient acceptance, AI aversion, survey experiment, generative AI

## Abstract

**Background:**

Artificial intelligence (AI) technologies are increasingly integrated into medical practice, with AI-assisted diagnosis showing promise. However, patient acceptance of AI-assisted diagnosis, compared with human-only procedures, remains understudied, especially in the wake of generative AI advancements such as ChatGPT.

**Objective:**

This research examines patient preferences for doctors using AI assistance versus those relying solely on human expertise. It also studies demographic, social, and experiential factors influencing these preferences.

**Methods:**

We conducted a preregistered 4-group randomized survey experiment among a national sample representative of the US population on several demographic benchmarks (n=1762). Participants viewed identical doctor profiles, with varying AI usage descriptions: no AI mention (control, n=421), explicit nonuse (No AI, n=435), moderate use (Moderate AI, n=481), and extensive use (Extensive AI, n=425). Respondents reported their tendency to seek help, trust in the doctor as a person and a professional, knowledge of AI, frequency of using AI in their daily lives, demographics, and partisan identification. We analyzed the results with ordinary least squares regression (controlling for sociodemographic factors), mediation analysis, and moderation analysis. We also explored the moderating effect of past AI experiences on the tendency to seek help and trust in the doctor.

**Results:**

Mentioning that the doctor uses AI to assist in diagnosis consistently decreased trust and intention to seek help. Trust and intention to seek help (measured with a 5-point Likert scale and coded as 0‐1 with equal intervals in between) were highest when AI was explicitly absent (control group: mean 0.50; No AI group: mean 0.63) and lowest when AI was extensively used (Extensive AI group: mean 0.30; Moderate AI group: mean 0.34). A linear regression controlling for demographics suggested that the negative effect of AI assistance was significant with a large effect size (β=−.45, 95% CI −0.49 to −0.40, *t*_1740_=−20.81; *P*<.001). This pattern was consistent for trust in the doctor as a person (β=−.33, 95% CI −0.37 to −0.28, *t*_1733_=−14.41; *P*<.001) and as a professional (β=−.40, 95% CI −0.45 to −0.36, *t*_1735_=−18.54; *P*<.001). Results were consistent across age, gender, education, and partisanship, indicating a broad aversion to AI-assisted diagnosis. Moderation analyses suggested that the “AI trust gap” shrank as AI use frequency increased (interaction term: β=.09, 95% CI 0.04-0.13, *t*_1735_=4.06; *P*<.001) but expanded as self-reported knowledge increased (interaction term: β=−.04, 95% CI −0.08 to 0.00, *t*_1736_=−1.75; *P*=.08).

**Conclusions:**

Despite AI’s growing role in medicine, patients still prefer human-only expertise, regardless of partisanship and demographics, underscoring the need for strategies to build trust in AI technologies in health care.

## Introduction

In the United States, the Food and Drug Administration has approved artificial intelligence (AI) use in various health care scenarios [1]. AI has facilitated medical processes by reducing decision-making errors, lowering costs, and speeding up diagnoses [2,3]. Despite these benefits, AI diagnosis faces psychological and structural barriers to patient acceptance. These include beliefs that AI cannot consider individual-specific factors [4], uncertainty about liability [5,6], and concerns about data privacy [2,6]. As a result, many consumers remain reluctant to accept fully automated diagnosis [2,4,7].

These challenges have led to a more moderate approach, AI-assisted diagnosis, where AI is a supportive tool for human doctors rather than a replacement [2,7]. Studies show that patients prefer this collaborative approach over fully automated systems [2,7,8]. For instance, Jutzi et al [9] found that 94% of patients would accept AI assistance in skin cancer screening, but this number shrank to 41% when AI stands alone. Palmisciano et al [10] revealed that the acceptance of fully automated neurosurgery was only 17.7% compared with 47.7% for AI-assisted neurosurgery. This is possibly because AI-assisted diagnosis combines the credibility of human expertise with the efficiency and cost-effectiveness of AI [2,11].

However, most prior research focused on earlier forms of AI, such as image readers [7,12], high-risk patient identifiers [13,14], and virtual assistant chatbots [15,16], and was conducted before the emergence of large language model–based generative AI. The emergence of generative AI has introduced a new dimension to medical AI [17]. Generative AI models excel in conducting clinical reasoning and helping medical education [18,19], diagnosing complex cases [3], and enabling text-to-protein generation [20]. These applications have marked a considerable paradigm change in medical AI because of their controllability, adaptability, and applicability [21].

Nonetheless, several concerns limit the public’s acceptance of generative models in health care. First, generative AI models can produce hallucinations, instances where they generate information that sounds plausible but is factually incorrect or entirely fabricated [22]. This is especially concerning because false diagnoses, incorrect treatment recommendations, or fabricated medical facts could seriously affect patients’ health and well-being. Moreover, because commercial firms develop most generative AI products, their lack of transparency and ethical risks to privacy and data protection are potential challenges [3]. Finally, as generative AI is still a relatively novel technology, the general public may have limited familiarity with it and may not yet associate it with medical applications, which could pose a barrier to its acceptability [23].

Because of these issues, whether patients accept generative AI as a tool to support diagnosis remains an open question that requires empirical exploration. To fill this gap, this research is interested in the extent to which people would prefer doctors using AI assistance to traditional doctors relying solely on human expertise. This research question is essential because understanding preferences for AI-assisted doctors can inform health care policy [24], guide the development of AI technologies in health care, and improve patient health outcomes [6] and satisfaction [25].

Previous research has applied the technology acceptance model (TAM) [26-28] and risk perception theory [29] to study the acceptance of AI-assisted diagnosis. Both theories suggest a cost-benefit analysis of perceived gains and hazards from the signals observed about AI-assisted diagnosis as a technology. These theories have implied several factors associated with the acceptance of AI-assisted diagnosis. First, trust is a crucial psychological determinant for AI acceptance, as users must believe the potential benefits and overcome initial skepticism or fear of harmful risks to begin engaging with and ultimately accepting new technological innovations such as AI [23,28,30-32]. For instance, a recent study by Choung et al [28] directly applies the TAM theory on AI acceptance and teases out trust as a robustly significant factor. Juravle et al [31] and Shevtsova et al [32] both demonstrated the importance of trust in the acceptance of AI-assisted medical care. The role of trust is consistent across many studies, and its determinants have been revealed to include socioethical considerations, technical and design features, and user characteristics [33,34].

The TAM also suggests that the perceived ease of use is a key factor for consumers to accept a new technology [26,35]. Familiarity, as predicted by intuition, breeds perceived ease of use [36,37]. Also, it reduces the concern of unexpected risks or harms, aligning with the risk perception theory. Familiarity with AI or new technology also matters because it may breed more comfort and trust in the technology adoption [23]. For instance, continued experience with AI-based algorithms may help develop trust over time [38]. These 2 pathways both determine the importance of technological familiarity.

Beyond the insights from TAM and risk perception theory, communication strategies also shape how people perceive both the benefits and potential downsides of AI [29,39,40]. When AI is framed as an adaptive entity capable of learning from user interactions, a humanized agent endowed with relatable traits (eg, conversational tone and empathy cues), and a collaborative partner designed to augment—rather than replace—human agency [8,11], users are more likely to perceive it as trustworthy and predictable.

Last but not least, user attributes are crucial determinants in AI trust and acceptance [34]. For instance, past studies have suggested that Republicans (conservatives) are typically more skeptical of science and technology [41], signaling a potentially stronger aversion to AI-assisted health care. Preliminary evidence supports this, showing an exceptionally high aversion to AI in diagnosis among conservatives [42,43], with some past research linking conservative and risk aversion to AI’s uncertainty [42].

This study examines an AI-assisted diagnostic approach designed to work alongside existing health care practices rather than function as a stand-alone tool. Patients are presented with a doctor who may or may not rely on AI for evaluating and diagnosing moderate symptoms. Because the study focuses on people’s general responses to the idea of AI in medical settings, it does not target a specific patient population or condition. We hypothesized that Republicans distrust AI-assisted health care more. However, we did not have a directional hypothesis regarding the general public’s perception of AI-assisted diagnosis. Additionally, we explored the impact of various demographic characteristics on AI acceptance.

## Methods

### Experimental Design and Data Sources

From June 17 to 20, 2024, we recruited 1762 participants via Prolific, a web-based subject platform suitable for survey and experimental research. This platform has a high response quality and a low dishonesty rate [44,45]. The sample represented the US population based on gender, age, and political affiliation.

**Figure 1.**
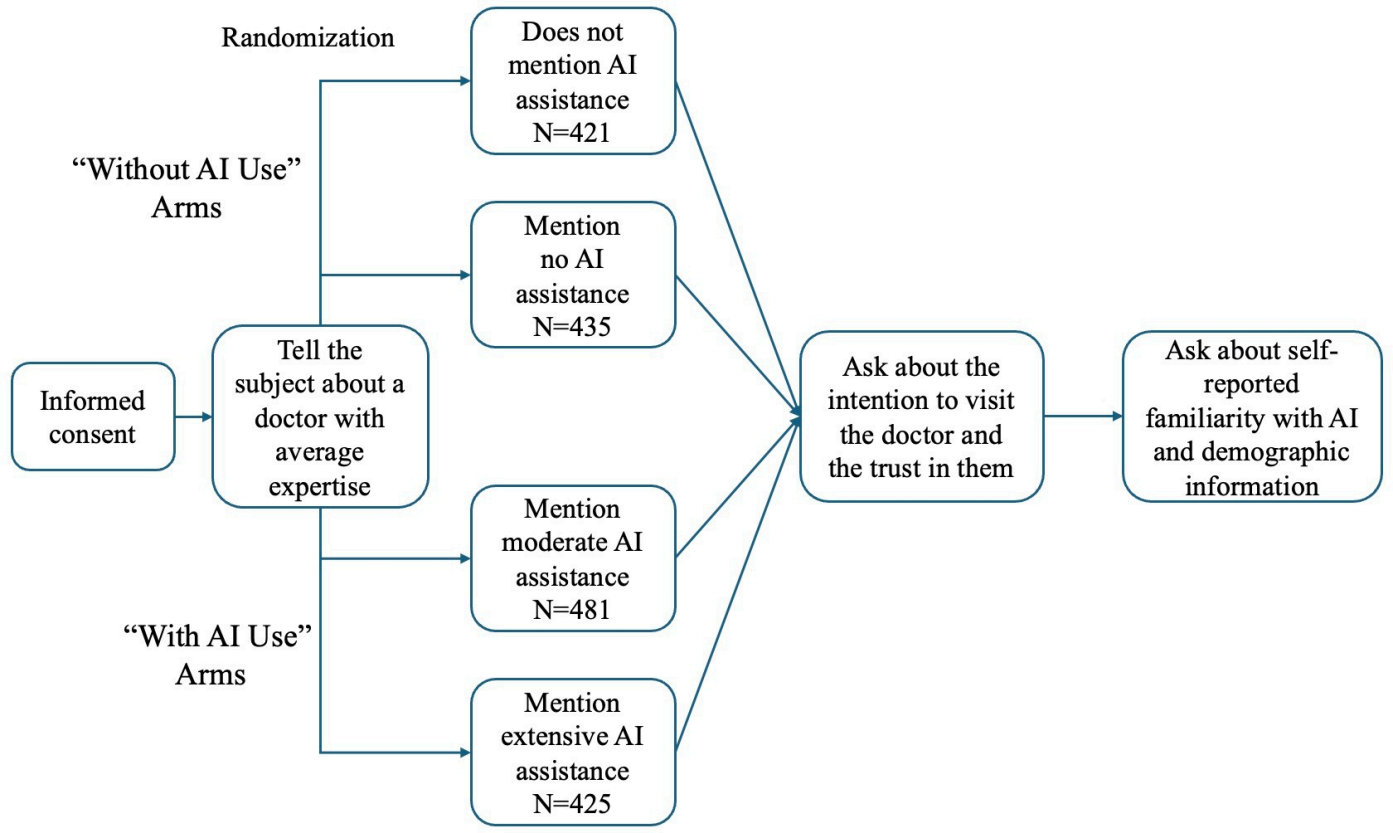
Flowchart of the experiment design. AI: artificial intelligence.

Participants were randomly assigned to 1 of 4 treatment arms. In all 4 groups, participants were presented with identical information about an illness and a typical doctor (“You’re having some moderate symptoms and are thinking about getting advice from a doctor. Here is Doctor M, a doctor with average experience and expertise in treating your illness.”). Figure 1 shows a flowchart illustrating the experimental design.

The control group did not mention AI-related content. In the “No AI” group, participants were informed that the doctor does not use AI assistance for diagnosis or treatment (“Doctor M doesn’t use artificial intelligence (AI, such as ChatGPT) at all when evaluating symptoms and deciding on treatments.”). These 2 groups are coded as “without AI use.” The “Moderate AI” group noted that the doctor uses AI, such as ChatGPT, to a moderate extent when evaluating symptoms and deciding on treatments. The information in the “High AI” group indicated that the doctor uses AI, such as ChatGPT, extensively when evaluating symptoms and deciding on treatments. These 2 groups are coded as “with AI use.”

Next, respondents reported their tendency to seek help, trust in the doctor as a person and a professional, knowledge of AI, and frequency of using AI in their daily lives. All these measures were measured from a 5-point Likert scale and coded into 0, 1/4, 1/2, 3/4, and 1. Then, we collected the participants’ demographics and partisan identification. The interface that a surveyee may face during the experiment is demonstrated in Multimedia Appendix 1. The left panel suggests a PC/Mac interface, and the right panel suggests a smartphone interface.

### Analysis of the Main Effect

Following the preregistration (OSF ID: v8kzs) [46], the main results were obtained through ordinary least squares (OLS) regression. We controlled for gender, age, income, education, race, and Hispanic ethnicity. Preregistered OLS regressions were performed on self-identified Republicans. The main regression is specified as follows:


(1) $$Y_{i}=\beta \times AI\_{Use}_{i}+{Controls}_{i}+u_{i}$$

where $Y_{i}$ is the outcome variable (trust as a professional or an individual, and the intention to seek help), and $AI\_{Use}_{i}$ is a binary variable with 1 indicating that the doctor uses AI-assisted diagnosis.

When examining the effects of AI use intensity within the Republican sample (as preregistered), we conducted the following regression analysis among participants who were assigned to an AI-assisted diagnosis condition:


(2) $$Y_{i}=\beta \times {Extensive}_{i}+{Controls}_{i}+u_{i}$$

where *${Extensive}_{i}$* is a binary variable with 1 indicating that the doctor uses AI-assisted diagnosis extensively and 0 indicating that the doctor uses AI assistance moderately. It was hypothesized that Republicans are less likely to seek help from doctors who use AI extensively compared with those who use AI moderately. We report the standardized OLS regression coefficients as indicators of the effect size [47].

The same regression analyses (1) and (2) were also conducted on the full sample as part of an exploratory analysis. While we did not preregister a directional hypothesis, examining the sign and magnitude of the effect in the general population remains valuable. Additionally, we assess how AI use impacts individuals across different demographic backgrounds.

### Moderation and Mediation Analysis

To test the hypothesis that Republican partisanship leads to a greater aversion to AI-assisted diagnosis, we conducted a moderation analysis within the full sample. This analysis examined whether partisanship moderates the effect of AI use on trust and the intention to seek help. Moderation was assessed by analyzing the coefficients of interaction terms [48]:


$$\begin{array}{l} Y_{i}=\beta_{1}\times AI\_Use_{i}+\beta_{2}\times Rep_{i}+\beta_{3}\\ \times \left(AI\_Use_{i}\times Rep_{i}\right)+Controls_{i}+u_{i} \end{array}$$

where $Y_{i}$ is the intention to seek help, trust as a person, or trust as a professional. We coded ${Rep}_{i}$ in three ways: (1) a binary variable indicating self-reported Republican partisanship (Republican=1; Democrat and Independent=0), (2) a factor with 3 levels (Democrat, Republican, and Independent), and (3) a continuous variable of self-identified partisanship (“Strong Republican”=1, “Not Strong Republican”=5/6, “Lean toward the Republican Party”=2/3, “Don’t lean either way”=1/2, “Lean toward the Democratic Party”=1/3, “Not Strong Democrat”=1/6, and “Strong Democrat”=0).

Then, we conducted a preregistered mediation analysis to test the hypotheses that trust as a professional or an individual mediates the treatment effect of AI use on the intention to seek help. Mediation analyses were performed with the R package (R Foundation for Statistical Computing) *mediation* [49]. We also controlled for gender, age, income, education, Hispanic ethnicity, and race in the mediation analysis.

Finally, we conducted an exploratory moderation analysis to test the effect of AI familiarity (frequency of use and self-reported AI knowledge) in the full sample. The regression function is:


$$Y_{i}=\beta_{1}\times AI\_{Use}_{i}+\beta_{2}\times {Fam}_{i}+\beta_{3}\times \left(AI\_{Use}_{i}\times {Fam}_{i}\right)+{Controls}_{i}+u_{i}$$

### Data, Materials, and Software Availability

Preregistration, data, and related analysis (including code files) are available in the Open Science Framework repository (#v8kzs). The complete text of the treatments and survey questions is shown in Multimedia Appendix 2.

### Ethical Considerations

This research was conducted under the approval of the University of California Los Angeles institutional review board (No. 24‐000594). Informed consent procedures were implemented in accordance with University of California Los Angeles institutional review board regulations and were administered through Prolific. All data were fully anonymized at the time of collection. No personally identifiable information was collected. Participant responses were stored and analyzed in deidentified format. Participants received compensation consistent with Prolific’s fair pay guidelines. Each participant was compensated at or above the equivalent of US $9.78 per hour, consistent with fair wage standards. No identifiable participant images or materials are included in this manuscript or multimedia appendices.

## Results

### Summary Statistics

Summary statistics of main outcome variables and covariates are listed in Table 1. Balanced tests are shown in Table S1 in Multimedia Appendix 2. Demographic characteristics are well balanced across groups, with gender, age, and political affiliation distributions generally reflecting national averages. See Multimedia Appendix 2 for demographic comparisons between the current sample and national statistics.

**Table 1.** Summary statistics of main outcome variables and covariates.

Variables	Summary statistics (n=1762)
Outcome variables, 0‐1 continuous scale, mean (SD)	
Intention to visit the doctor	0.44 (0.28)
Trust of the doctor as a person	0.47 (0.23)
Trust of the doctor as a professional	0.48 (0.26)
Group allocation, n (%)	
Does not mention AI^a^	421 (23.9)
Mention no AI use	435 (24.7)
Moderate AI use	481 (27.3)
Extensive AI use	425 (24.1)
Demographics	
Female, n (%)	916 (52.0)
Age (years), n (%)	
18‐29	312 (17.7)
30‐39	335 (19.0)
40‐49	278 (15.8)
50‐59	384 (21.8)
60‐69	337 (19.1)
≥70	116 (6.6)
Mean age (SD), years	47.0 (15.7)
Race, n (%)	
White	1455 (82.6)
Black	180 (10.2)
Asian	129 (7.3)
Native American	32 (1.8)
Hispanic	141 (8.0)
Other	34 (1.9)
Annual income cohort, n (%)	
<20,000	152 (8.6)
20,000‐50,000	422 (24.0)
50,000‐75,000	339 (19.2)
75,000-100,000	305 (17.3)
>100,000	544 (30.9)
Education level, n (%)	
Less than high school	14 (0.8)
High school graduate	189 (10.7)
Some college/associate	562 (31.9)
College graduate	657 (37.3)
Postgraduate	340 (19.3)
Partisanship identity, n (%)	
Strong Democrat	350 (19.9)
Democrat	208 (11.8)
Independent leaning Democrat	297 (16.9)
Independent	214 (12.2)
Independent leaning Republican	178 (10.1)
Republican	264 (15.0)
Strong Republican	251 (14.3)
Continuous partisan score (SD)	0.47 (0.35)
Self-identity as Republican, n (%)	515 (29.2)
AI use experience, n (%)	
Not frequently at all	578 (32.9)
Slightly frequently	555 (31.6)
Moderately frequently	330 (18.8)
Very frequently	211 (12.0)
Extremely frequently	85 (4.8)
Continuous AI use score, mean (SD)	0.31 (0.29)
Self-reported AI knowledge level, n (%)	
None at all	49 (2.8)
A little	476 (27.1)
A moderate amount	739 (42.0)
A lot	358 (20.3)
A great deal	138 (7.8)
Continuous knowledge score, mean (SD)	0.51 (0.24)

a AI: artificial intelligence.

### Main Effect

**Figure 2.**
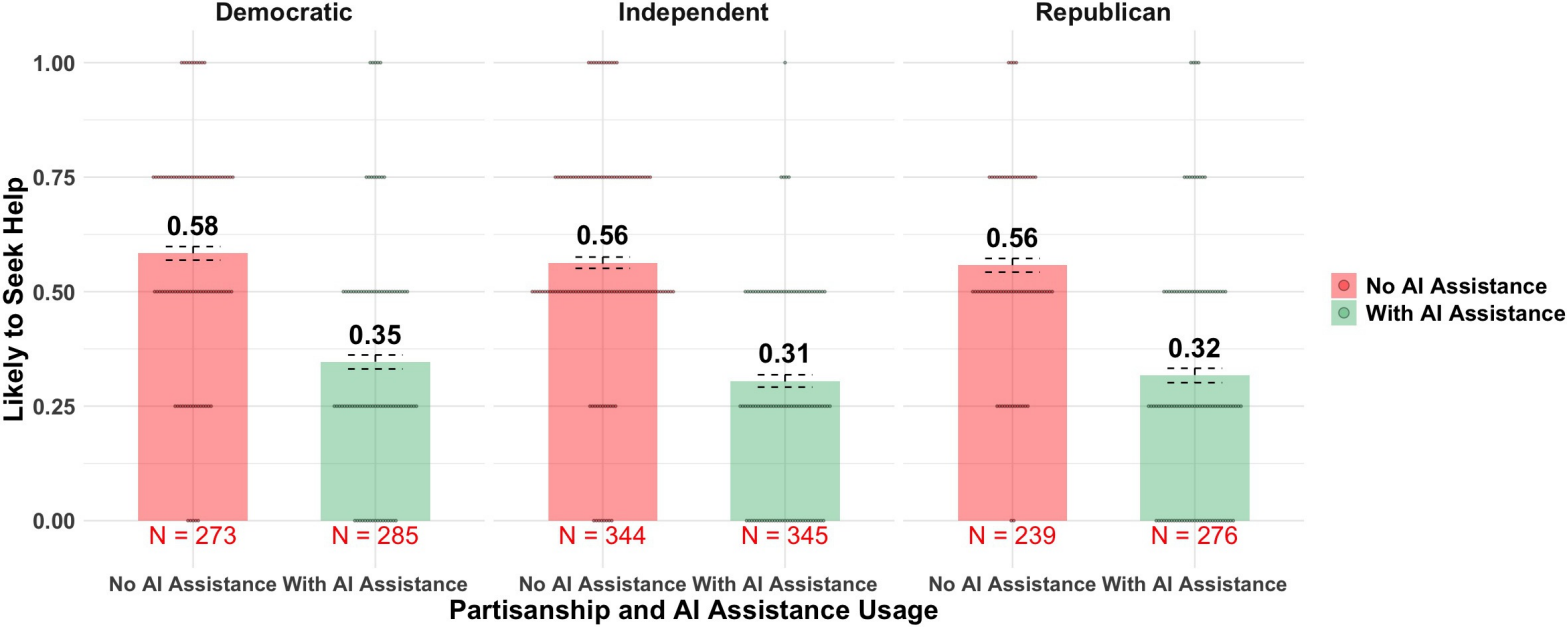
The effect of doctor’s AI usage on patients’ intention to seek help in different partisan groups. Error bars represent SE. AI: artificial intelligence.

Figure 2 shows the effect of doctor’s AI usage on patients’ intention to seek help in different partisan groups. Among Republicans, the average intention to seek help is 0.56 in groups where the doctor does not use AI, but it drops to 0.32 in groups where the doctor uses AI. Consistent with our hypothesis, mentioning a doctor’s use of AI reduced respondents’ trust in the doctor (as a person: β=−.31, 95% CI −0.39 to −0.22, *t*_491_=−7.20, *P*<.001, adjusted *R*^2^=0.11; as a professional: β=−.38, 95% CI −0.46 to −0.30, *t*_491_=−9.28, *P*<.001, adjusted *R*^2^=0.13) and decreased respondents’ intention to seek help (β=−.43, 95% CI −0.51 to −0.35, *t*_494_=−10.58, *P*<.001, adjusted *R*^2^=0.19).

**Figure 3.**
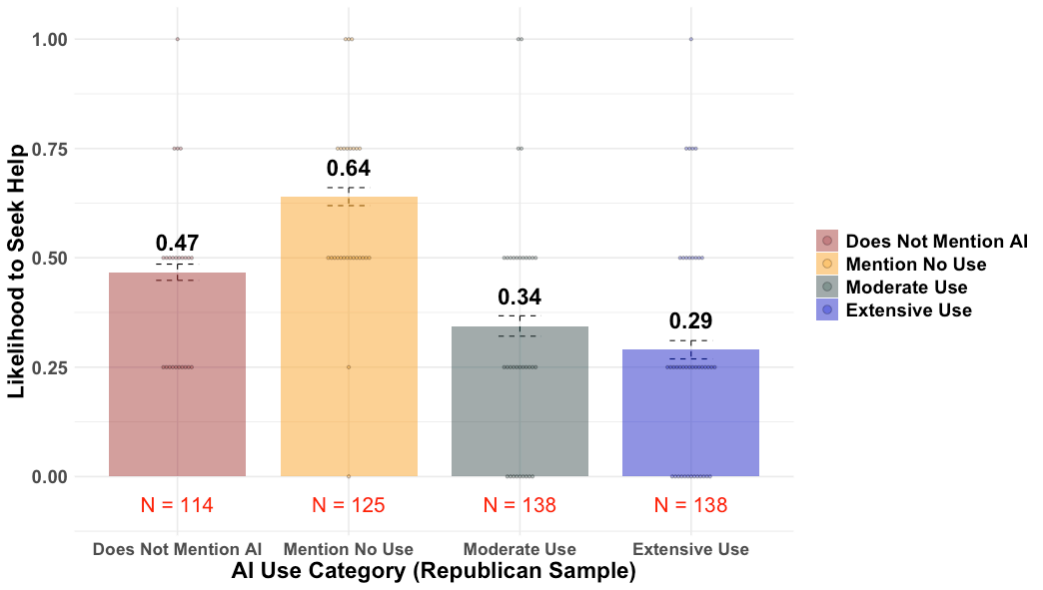
Republican patients’ intention to seek help in all 4 treatment groups. Error bars represent SE. AI: artificial intelligence.

We also preregistered that Republicans would be less likely to seek help from doctors who use AI extensively than from those who use AI moderately. However, as demonstrated in Figure 3 (Moderate Use group: mean 0.34; Extensive Use group: mean 0.29), the intention to seek help did not differ significantly among Republicans, regardless of whether the doctor used AI extensively or moderately (β=.20, 95% CI −0.04 to 0.45, *t*_255_=1.64, *P*=.102, adjusted *R*^2^=0.006). Similarly, Republicans’ trust in the doctor, both as a person and as a professional, did not differ significantly based on the extent of AI use (as a person: β=.19, 95% CI −0.05 to 0.42, *t*_254_=1.56, *P*=.12, adjusted *R*^2^=0.006; as a professional: β=.23, 95% CI −0.01 to 0.47, *t*_254_=1.88, *P*=.06, adjusted *R*^2^=0.032).

**Figure 4.**
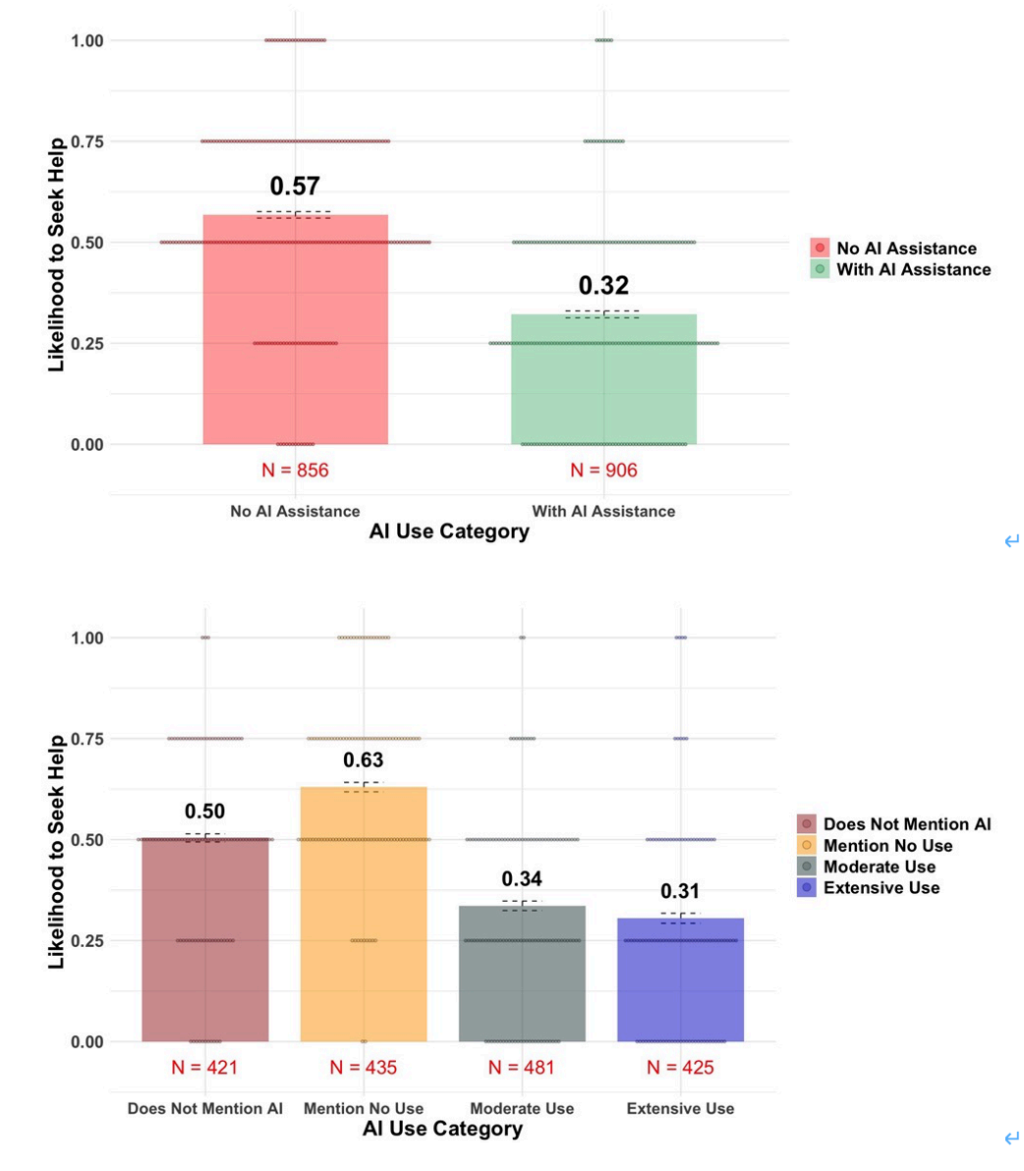
Patients’ intention to seek help under different treatment conditions (full sample, by AI use status, and by treatment condition). Error bars represent SE. AI: artificial intelligence.

Next, we conducted exploratory analyses using the full sample. The 2 panels in Figure 4 show the effect of the doctor’s AI use on a patient’s visit intention in the full sample. Similarly to what we found among Republicans, across the entire sample, AI use reduced trust (as a person: β=−.33, 95% CI −0.37 to −0.28, *t*_1733_=−14.41, *P*<.001, adjusted *R*^2^=0.20; as a professional: β=−0.40, 95% CI −0.37 to −0.28, *t*_1735_=−18.54, *P*<.001, adjusted *R*^2^=0.11) and decreased respondents’ intention to seek help (β=−0.45, 95% CI −0.45 to −0.36, *t*_1740_=−20.81, *P*<.001, adjusted *R*^2^=0.17).

Interestingly, respondents’ trust in the doctor and their intention to seek help were highest when the prompt explicitly stated that the doctor does not use AI at all in their diagnosis (control group: mean 0.50; No AI group: mean 0.63; Extensive AI group: mean 0.31; and Moderate AI group: mean 0.34). Taken together, Figures 2-4 suggest that the aversion to AI assistance was consistent across party identifications.

**Figure 5.**
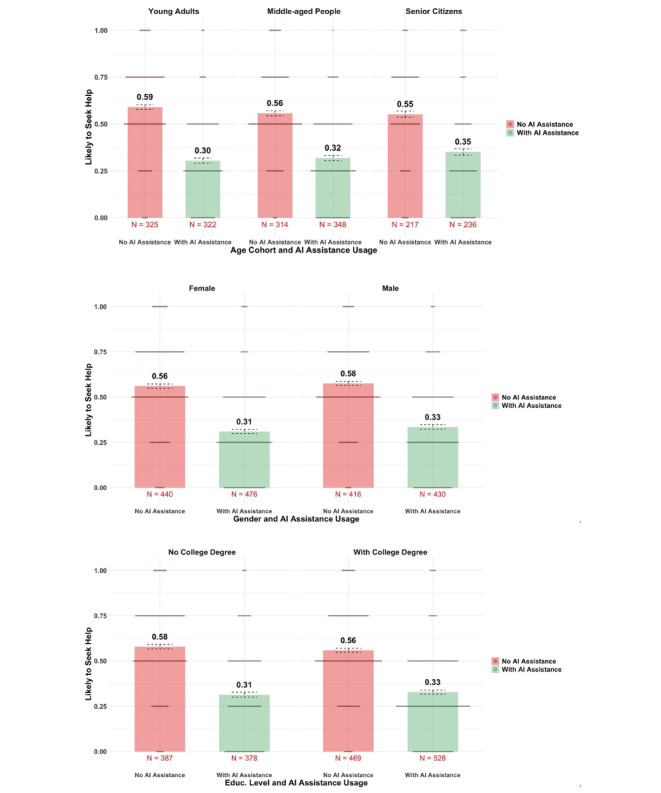
Intention to seek help among patients from different demographic backgrounds (full sample). Error bars represent SE. AI: artificial intelligence.

Moreover, the 3 panels of Figure 5 further showed that the aversion to AI assistance was consistent across age, gender, and education level. The distrust was distributed evenly across the population.

As another robustness check, we tested 3-way interactions (the treatment condition interacted with 2 demographic and political variables) to examine whether the treatment effects varied across intersecting subgroup combinations. We ran 10 ANOVA tests on all 10 combinations of 5 demographic and political variables (age, gender, income, education level, and partisanship). None of the 3-way interactions was significant at the .05 level. This adds further evidence to our argument that the AI aversion is uniform.

### Moderation and Mediation

We preregistered a moderation analysis to test whether Republican partisanship negatively moderates the effect of AI use on the intention to seek help. However, Republican partisanship did not have a significant moderating effect. This is supported by the interaction regression with 3 different specifications of Republican partisanship. The coefficient of the interaction term is not significant regardless of how partisanship was coded (binary variable: β=.01, *t*_1738_=0.34, *P*=.73; 3-level factor: β=−.01, *t*_1736_=−0.18, *P*=.86; and continuous variable: β=.001, *t*_1738_=0.07, *P*=.94). The aversion to AI assistance was consistent across party identification.

We preregistered that the effect of AI usage on visiting intention is mediated by trust in the doctor. This was supported both among Republicans (mediation by the trust as a person: average causal mediation effect [ACME]=−0.11, *P*<.001, average direct effect [ADE]=−0.12, *P*<.001; mediation by the trust as a professional: ACME=−0.16, *P*<.001, ADE=−0.08, *P*<.001) and among the full sample (mediation by trust as a person: ACME=−0.11, *P*<.001, ADE=−0.13, *P*<.001; mediation by trust as a professional: ACME=−0.17, *P*<.001, ADE=−0.08, *P*<.001).

**Figure 6.**
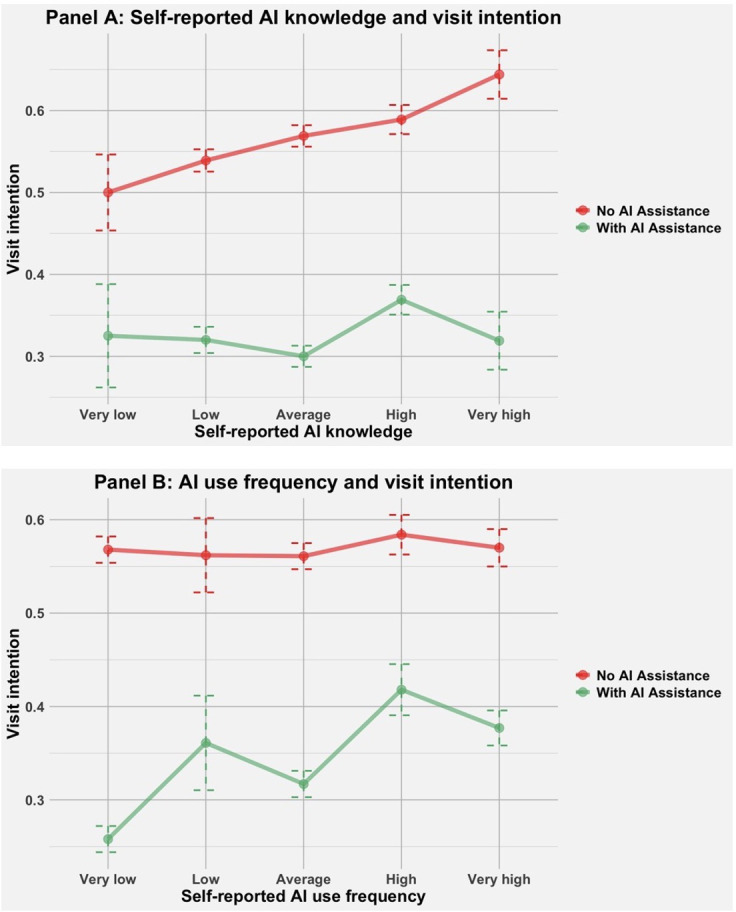
Panel A: The impact of a doctor’s AI usage on a patient’s visit intention was moderated by the patient’s self-reported AI knowledge. Panel B: The impact of a doctor’s AI usage on a patient’s visit intention was moderated by the patient’s self-reported AI use frequency. AI: artificial intelligence.

Surprisingly, despite the high correlation between AI knowledge and AI use frequency (*r*=0.61), these 2 variables had opposite moderating effects (Figures 6A and B). In the entire sample, the negative effect of AI assistance diminished as participants’ AI use frequency increased (interaction term β=.09, 95% CI 0.04 to 0.13, *t*_1735_=4.06; *P*<.001). In other words, the “trust gap” was smallest among those who used AI most frequently. Conversely, the “trust gap” expanded as participants’ self-reported AI knowledge increased, with interaction term β=−.04, 95% CI −0.08 to 0.00, *t*_1736_=−1.75; *P*=.08. Participants who reported the highest AI knowledge exhibited the largest trust gap.

## Discussion

### Summary of Findings

This study investigated the impact of AI-assisted diagnosis on patients’ trust in doctors and their intention to seek medical help. Mentioning a doctor’s use of AI significantly decreased respondents’ trust in the doctor, both as a person and as a professional, and in turn, reduced their intention to seek help (partial mediation in both Republicans and the full sample).

We preregistered that Republicans would be more averse to AI-assisted diagnosis because previous literature suggested that conservatives are more likely to resist changes and new technology [41,43]. However, our results revealed that Americans’ distrust of AI-assisted diagnosis surpassed partisanship and demographic lines. The distrust was consistent across age, gender, education, and party identification. In other words, Americans’ distrust of AI-assisted diagnosis is general rather than a conservatism-specific phenomenon. Furthermore, trust in the doctor and the intention to seek help were highest when the doctor explicitly renounced AI in their diagnosis, suggesting that patients may still prefer human-only expertise in medical care.

### Why Is the Aversion Significant and Widespread?

A major finding of this paper is the significant and widespread aversion toward AI-assisted diagnosis in the United States. This result deviates from previous research on specific AI health care products [2,8]. We propose 2 potential explanations for this discrepancy.

First, generative AI systems differ fundamentally from earlier AI health care products, such as image recognition [14,50] and wearable devices [51]. These legacy tools operate within predictable parameters (eg, identifying tumors and tracking heart rates), while generative AI engages in more open-ended interactions that blur the human-machine boundary. Prior AI health care products have already achieved some maturity through years of public exposure, and people may already be familiar with them.

Second, ChatGPT and other large language models are mostly general purpose AI. Different from AI models specifically trained for medical purposes, these models are not intended to be used in clinical scenarios without human oversight [52]. Given their broad applicability and direct accessibility, patients may question the professionalism or judgment of doctors who use such tools. This skepticism may be a result of valid concerns: there is evidence that AI-assisted teams may perform inefficiently because of cognitive challenges such as overreliance on the AI [53] and the lack of metaknowledge [54]. This finding may extend to other nonmedical, unfamiliar technologies: when tools lack a clear medical focus and public familiarity, their integration into health care may lower patient acceptance and satisfaction.

### Why Do AI Knowledge and AI Experience Shape Trust Differently?

As an exploratory analysis, we found that the “trust gap” was smallest among those who used AI most frequently. One explanation is that regular AI use fosters comfort and familiarity [14,23], which, in turn, enhances trust in AI-assisted health care [55,56]. Previous literature has indicated that familiarity increases perceived ease of use [35,38] and thus increases support for the adoption of AI in areas including autonomous systems [23], retail chatbots [55], and travel planning [56,57].

Yet, participants who reported the highest AI knowledge exhibited the largest trust gap. This finding seems counterintuitive at first glance because greater knowledge of technology is typically associated with increased understanding and trust, rather than heightened skepticism [58]. Nonetheless, recent studies [23] do suggest that “knowledge” does not correlate with acceptance in AI systems as “using experience” does. While this finding provides some support for our results, it does not fully explain why we observed a negative correlation. To address this, we propose the following reasons why the negative effect of AI assistance expanded as self-reported AI knowledge increased.

First, a potential channel is that the higher the self-reported AI knowledge, the more aware respondents were of generative AI’s limitations, risks, and ethical concerns [23]. As general AI is not specifically designed for diagnostics, those with greater awareness may distrust its use in high-stakes contexts. This heightened awareness can amplify concerns about reliability, accuracy, and maturity in medical settings.

Second, the awareness of AI’s limitations may stem from *perceived* rather than *actual* risks. People confident in their belief that health care AI is inadequate may misunderstand its true capabilities. Common concerns, such as AI’s rigidity and inability to personalize care, often drive aversion [4,39], although AI can sometimes outperform humans in these areas [3,19]. Those who strongly believe in these misconceptions may report high AI knowledge while simultaneously exhibiting AI aversion.

There is also the possibility of overconfidence, given respondents self-reported their perceived level of AI knowledge [59,60]. For instance, recent research on women’s attitudes toward medical AI [61] found that respondents with lower educational levels (which correlates with less real knowledge) reported a higher self-perceived accuracy of AI knowledge, consistent with our overconfidence hypothesis. Similarly, studies on health literacy suggested that individuals with higher self-reported knowledge may possess lower actual literacy [60,62]. These patterns align with our findings, as self-reported AI knowledge may not reflect true AI literacy but rather an overestimation of one’s understanding coupled with an underestimation of AI’s capabilities. Such overconfidence can lead to an exaggerated perception of AI’s weaknesses, contributing to the observed trust gap. Unlike self-reported knowledge, experience reflects real interactions (eg, using AI at work or in daily life). This practical experience likely offers a more accurate view of AI’s strengths and weaknesses, which may explain why frequent users showed less aversion.

### Theoretical Contributions

This research has the following theoretical implications. First, we extended previous literature on medical AI aversion [4,39,40] and found that aversion widely persists even if AI is used as an *assisting tool* rather than a sole decision maker in the era of generative AI. We also found that the aversion to AI in health care is not confined to any specific demographic or ideological group, suggesting that concerns about AI-driven diagnoses are broadly held rather than politically polarized.

Second, we challenge assumptions about the role of AI familiarity in mitigating skepticism. While prior studies suggest that increased exposure to AI enhances trust and we obtained similar results, we found that self-reported AI knowledge widened the trust gap, whereas frequent AI use narrowed it. The proposed reasons in the previous section contribute to theories of algorithmic trust, highlighting differential roles of perceived expertise (knowledge) versus experiential familiarity. This necessitates better measures for AI familiarity.

Third, our findings contribute to the literature on trust and decision-making. Trust in the doctor’s personal integrity and professional competence are key mediators of AI-assistance aversion. In our case, trust acts as a *barrier* to AI-assisted health care adoption, as lower trust in doctors who use AI directly reduces patients’ intention to seek medical help. Trust calibration (ensuring alignment between perceived and actual AI reliability) remains a challenge for medical AI integration.

### Practical Implications

The findings of this study offer important practical insights for health care professionals, policy makers, and AI developers working to integrate AI into decision-making.

First, we found that distrust and aversion appear when patients know that the doctor uses AI (such as ChatGPT), even if it serves as a supporting tool rather than a primary decision maker. Therefore, merely incorporating AI into medical diagnoses without solid trust-building ethics may inadvertently reduce patient’s trust. Understanding and addressing this trust barrier is essential for ensuring the successful adoption of AI-assisted health care.

Second, our results highlight the possible need for differentiating between self-reported AI knowledge and AI experience when designing patient education programs. Hands-on experiences with AI can demystify AI and increase trust and visiting intention [38]. Conversely, people with higher self-reported knowledge of AI showed larger gaps in trust and visiting intention. If individuals distrust AI based on self-perceived (but possibly inaccurate) knowledge, traditional knowledge-based educational interventions, especially if coming from sources already viewed with skepticism, may backfire. Communication strategies by governments and medical practitioners should be carefully designed to account for these biases, potentially incorporating experiential learning opportunities rather than relying solely on informational campaigns.

Third, we found that trust in the doctor and intention to seek help were highest when patients knew that the doctor does not use AI. This highlights the need for AI transparency in clinical settings.

### Limitations and Future Directions

One limitation of this study is sample selection. First, it used data only from the United States. Due to differences in economic development and culture, findings from the United States may not fully apply to other countries. Factors influencing trust in AI-assisted health care could vary across regions, and similar patterns of AI skepticism may not hold in different social environments [63] and cultural backgrounds [63-66]. Future research should examine cross-national patterns of AI acceptance.

Second, the Prolific sample may not reflect the general population, as its users are more inclined to use web-based services. However, we argue that in this study, this distortion is less problematic. Time spent online is positively associated with openness to experience [67,68], a trait linked to greater acceptance of AI [69]. Therefore, our estimate of AI aversion may represent a lower bound for the general population.

A recent study by the University of Queensland, covering 17 countries [64], found that US attitudes toward AI are similar to those in other high-income nations but more skeptical than in countries such as China, India, and South Africa. This supports partial generalizability to advanced health systems but calls for further research in transitional contexts.

We also used ChatGPT only as a demonstration for AI-assisted diagnostic tools. It is valuable to study different forms of AI assistance, especially those with growing popularity in recent years. Also, a clear comparison between generative AI and other categories of medical AI is pending further exploration.

This study did not investigate ethical design and transparent communication strategies; yet, a successful integration of AI into health care requires attention to those aspects. Prior literature [40] suggested 5 possible strategies to improve AI acceptance: using explainable AI, anthropomorphizing, advertising AI as flexible, restoring user control, and convincing users of AI’s human-like consciousness. Without applying these strategies, AI-assisted diagnosis, particularly involving general purpose tools such as ChatGPT, may receive less acceptance. Future studies should explore how ethical framing, design transparency, and tailored communication can jointly reduce distrust and support safe AI adoption.

### Conclusions

Our research found a common aversion to AI-assisted diagnosis, especially within the domain of generative AI, and this aversion does not significantly vary across demographic and ideological cohorts. This highlights the potential challenges to the application of AI-assisted health care. We find that despite the benefits of AI in health care, there is still a strong preference for human expertise, which could slow the adoption of AI-assisted tools.

## Supplementary material

10.2196/66083 Multimedia Appendix 1 A Screenshot of the intervention. The left panel indicates the screen seen by a PC user, and the right panel indicates the screen seen by a smartphone user.

10.2196/66083 Multimedia Appendix 2 Extended results and methods.

10.2196/66083 Checklist 1 CONSORT-eHEALTH checklist (V 1.6.1).

## Abbreviations

ACME: average causal mediation effect
ADE: average direct effect
AI: artificial intelligence
OLS: ordinary least squares
TAM: technology acceptance model
