# Supplementary figures and images for: Impact of AI-Assisted Diagnosis on American Patients’ Trust in and Intention to Seek Help From Health Care Professionals: Randomized, Web-Based Survey Experiment
Source: J Med Internet Res. 2025 Jun 18;27:e66083. doi: 10.2196/66083 (PMC12222559; doi:10.2196/66083)

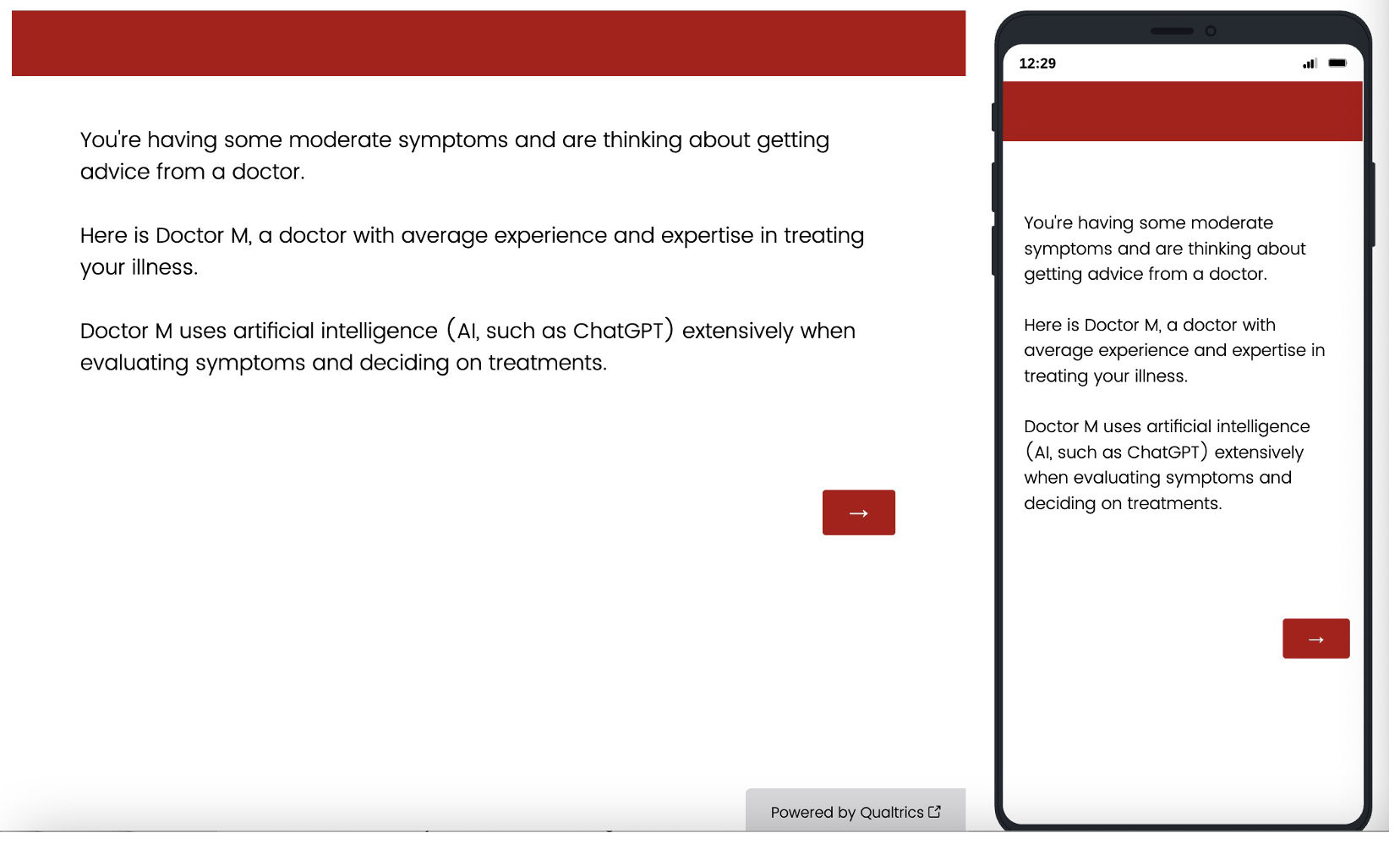

Supplement: Multimedia Appendix 1 [file jmir-v27-e66083-s001.png]
