# Supplementary material for: Impact of AI-Assisted Diagnosis on American Patients’ Trust in and Intention to Seek Help From Health Care Professionals: Randomized, Web-Based Survey Experiment
Source: J Med Internet Res. 2025 Jun 18;27:e66083. doi: 10.2196/66083 (PMC12222559; doi:10.2196/66083)
Supplement: Multimedia Appendix 2 [file jmir-v27-e66083-s002.docx]

| Table S1. Balance tests of treatment groups indicating the doctor uses or does not use AI. | | | |
| --- | --- | --- | --- |
|  | Use AI (n=856) | No AI (n=906) | Balance test (*P* value) |
| **Outcome and Mediator Variables** |  |  |  |
| Intention to visit the doctor | 0.57(0.24) | 0.32(0.26) | / |
| Trust of the doctor as a person | 0.55(0.21) | 0.40(0.23) | / |
| Trust of the doctor as a professional | 0.59(0.22) | 0.38(0.55) | / |
| **Demographics** |  |  |  |
| Female, n(%) | 440(51.4%) | 476(52.5%) | 0.633 |
| **Age, n(%)** |  |  |  |
| 18-29 | 152(17.8%) | 160(17.7%) | 0.957 |
| 30-39 | 173(20.2%) | 162(17.9%) | 0.213 |
| 40-49 | 131(15.3%) | 147(16.2%) | 0.596 |
| 50-59 | 183(21.4%) | 201(22.2%) | 0.682 |
| 60-69 | 159(18.6%) | 178(19.7%) | 0.567 |
| 70 or above | 58(6.78%) | 58(6.40%) | 0.752 |
| Mean age (SD) | 46.8(15.7%) | 47.2(15.8%) | 0.597 |
| **Race, n(%)** |  |  |  |
| White | 710(82.9%) | 745(82.2%) | 0.693 |
| Black | 88(10.3%) | 92(10.2%) | 0.931 |
| Asian | 67(7.8%) | 62(6.8%) | 0.428 |
| Native American | 17(2.0%) | 15(1.7%) | 0.604 |
| Other | 14(1.6%) | 20(2.2%) | 0.385 |
| **Hispanic** | 63(7.4) | 78(8.6) | 0.334 |
| **Annual Income Cohort, n(%)** |  |  |  |
| Less than 20K | 80(9.4%) | 72(8.0%) | 0.296 |
| 20-50K | 195(22.8%) | 227(25.1%) | 0.264 |
| 50-75K | 165(19.3%) | 174(19.2%) | 0.970 |
| 75K-100K | 147(17.2%) | 158(17.4%) | 0.883 |
| Above 100K | 269(31.4%) | 275(30.4%) | 0.626 |
| **Education Level, n(%)** |  |  |  |
| Less than High School | 6(0.7%) | 8(0.9%) | 0.668 |
| High School Graduate | 93(10.9%) | 96(10.6%) | 0.856 |
| Some College/Associate | 288(33.6%) | 274(30.2%) | 0.126 |
| College Grad | 310(36.2%) | 347(38.3%) | 0.366 |
| Postgrad | 159(18.6%) | 181(20.0%) | 0.456 |
| **Partisanship Identity, n(%)** |  |  |  |
| Strong Democrat (0) | 159(18.6%) | 191(21.1%) | 0.188 |
| Democrat (1/6) | 114(13.3%) | 94(10.4%) | 0.056 |
| Independent Leaning Democrat (1/3) | 152(17.8%) | 145(16.0%) | 0.326 |
| Independent (1/2) | 112(13.1%) | 102(11.3%) | 0.241 |
| Independent Leaning Republican (2/3) | 80(9.4%) | 98(10.8%) | 0.306 |
| Republican (5/6) | 130(15.2%) | 134(14.8%) | 0.816 |
| Strong Republican (1) | 109(12.7%) | 142(15.7%) | 0.078 |
| Continuous Partisan Score (SD) | 0.46(0.34) | 0.479(0.36) | 0.336 |
| Self-identity as Republican, n(%) | 239(27.9%) | 276(30.5%) | 0.241 |
| **AI Use Experience, n(%)** |  |  |  |
| Not frequently at all | 276(32.3%) | 302(33.4%) | 0.626 |
| Slightly frequently | 261(30.6%) | 294(32.5%) | 0.376 |
| Moderately frequently | 161(18.9%) | 169(18.7%) | 0.934 |
| Very frequently | 116(13.6%) | 95(10.5%) | 0.048* |
| Extremely frequently | 40(4.7%) | 45(5.0%) | 0.773 |
| Continuous AI Use Score (SD) | 0.32(0.30) | 0.30(0.29) | 0.242 |
| **Self-reported AI Knowledge Level** |  |  |  |
| None at all | 29(3.4%) | 20(2.2%) | 0.135 |
| A little | 233(27.2%) | 243(26.9%) | 0.851 |
| A moderate amount | 356(41.6%) | 383(42.3%) | 0.771 |
| A lot | 171(20.0%) | 187(20.7%) | 0.729 |
| A great deal | 66(7.7%) | 72(8.0%) | 0.853 |
| Continuous Knowledge Score (SD) | 0.50(0.24) | 0.51(0.23) | 0.388 |

(1) The average female percentage is 52%, similar to the 50.5% national average.

(2) The median age of the sample is 47.0, which is very close to the national average age (47.3) in 2023 among the ADULT population. The compositions of age cohorts from 18-29 to 60-69 are also similar to the national average. However, due to the fact that people over 70 are much less likely to use Internet, the proportion is significantly lower than the national average. This is unlikely to distort our findings because these people are much less to be Pro-AI, which indicates that our findings of AI aversion are still valid.

(3) The partisanship distribution is similar to the national average. Here, about 32% identified themselves as Democrats, 39% as Independents and 29% as Republicans. This is similar to the 2020 distribution recorded by Gallup [49]: 30% Independents, 39% Independents and 29% Republicans (2% Missing).

| Table S2. Three-way interaction ANOVA tests for uniformaliy of AI aversion. | | | | | |
| --- | --- | --- | --- | --- | --- |
| **Variable** | Age | Female | Education level | Income level | Partisanship  (Rep/Dem/Ind) |
| Age |  | F(5,1737)=1.35  *P*=.24  $\eta^{2}=0.004$ | F(16,1706)=1.35  *P*=.16  $\eta^{2}=0.01$ | F(20,1701)=0.88  *P*=.88  $\eta^{2}=0.01$ | F(10,1725)=1.25  *P*=.25  $\eta^{2}=0.007$ |
| Gender |  |  | F(4,1741)=1.29  *P*=.27  $\eta^{2}=0.003$ | F(4,1741)=0.74  *P*=.57  $\eta^{2}=0.003$ | F(2,1749)=0.97  *P*=.38  $\eta^{2}=0.001$ |
| Education level |  |  |  | F(14,1713)=0.67  *P*=.80  $\eta^{2}=0.005$ | F(7,1732)=0.77  *P*=.61  $\eta^{2}=0.003$ |
| Income level |  |  |  |  | F(8,1731)=0.75  *P*=.65  $\eta^{2}=0.003$ |
| Partisanship  (Rep/Dem/Ind) |  |  |  |  |  |
| *p<0.05. **p<0.01. ***p<0.001. | | | | |  |

Among all ANOVA tests for major demographic and political affiliation variables, no specifc interactions generated significant results.

**Supporting Information Text**

**Details of measurements**

*Manipulated variables.*

The only manipulated variable was the group assignment of the information about the doctor's use of AI-assisted diagnosis.

Specifically, in the control group, participants saw information about a doctor (Doctor M) without any mention of AI-related content. In the "No AI" group, the doctor's information mentioned that the doctor does not use AI assistance for diagnosis and treatment. In the "Moderate AI" group, it was mentioned that the doctor uses AI at a moderate intensity. In the "High AI" group, the information indicated that the doctor uses AI at a high intensity. Following this, we asked participants about their inclination to visit this doctor for medical advice, as well as their trust in the doctor.

*Measured variables.*

Treatment Conditions: We labeled each treatment group as a categorical variable. The "Doctor uses AI extensively" group was Category 1, the "Doctor uses AI moderately" group was Category 2, the "Doctor does not use AI" group was Category 3, and the "Does not mention AI" group was Category 4.

AI Use: Categories 1 and 2 was coded as 1, and Categories 3 and 4 was coded as 0.

AI Use Intensity: This variable only applied to Categories 1 and 2. Category 1 was coded as 1, and Category 2 was coded as 0.

Seek Help: Measured with a five-point Likert Scale question: “How likely are you to seek medical help from Doctor M?” The response options were “Not likely at all”, "Slightly likely", "Moderately likely", "Very likely", and "Extremely likely," and was coded as 0, 0.25, 0.5, 0.75, and 1 respectively.

Trust as a Person: Measured with a five-point Likert Scale question: "To what extent do you trust Doctor M as a person?" The response options were "None at all", "A little", "A moderate amount", "A lot", and "A great deal," and were coded as 0, 0.25, 0.5, 0.75, and 1 respectively.

Trust as a Professional: Measured with a five-point Likert Scale question: "To what extent do you trust Doctor M as a professional?" The options and coding were the same as above.

{Seek Help, Trust as a person, Trust as a Professional} appeared in random order.

Knowledge about AI: Measured with a five-point Likert Scale question: "How much do you know about AI, such as ChatGPT?" The options and coding were the same as above.

Frequency of AI Use: Measured with a five-point Likert Scale question: "Over the last six months, how frequently have you used AI, such as ChatGPT?" The response options were "Extremely frequently", “Very frequently”, "Moderately frequently", "Slightly frequently", and "Not at all frequently," and were coded as 1 to zero with equal intervals.

Female: Participants were asked, “What is your biological sex?” A dummy variable was created so that male respondents were coded as 0, and female respondents were coded as 1.

Age: Participants were asked, “In what year were you born?” Their age was calculated and coded into a factor with six levels: [18, 29], (29,39], (39,49], (49,59], (59,69], and above 69.

Education: Participants were asked, “What is the highest level of school you have completed or the highest degree you have received?” Responses were be coded as a factor with five levels: “Less than high school,” “High school graduate,” “Some college/Associate degree,” “College graduate,” and “Post-graduate”.

Income: Participants were asked to identify the total income for themselves and all members of their family who lived with them during the previous year before taxes. Responses were coded into a factor with five levels: “Less than 20k,” “20k to 50k,” “50k to 75k,” “75k to 100k,” and “above 100k”.

Ethnicity: Respondents were asked, “Are you of Hispanic, Latino, or Spanish origin?” A variable, “Hispanic,” was created and coded 1 when the participant answered “Yes” and 0 when not.

Race: Respondents were asked if they consider themselves to be from the following list: White, Black or African American, American Indian or Alaska Native, Asian, and Native Hawaiian or Pacific Islander. A dummy variable was be created for each response option and coded 1 if the respondent selects that option. (There are no respondents identifying as Native Hawaiian or Pacific Islander in our sample so we did not create the Native Hawaiian or Pacific Islander dummy.)

Party Identification: Respondents were randomly assigned to see one of the two versions of the party identification question: "Do you consider yourself a Democrat, a Republican, an Independent, or what?" or "Do you consider yourself a Republican, a Democrat, an Independent, or what?" Responses were coded as a categorical variable with three levels: Democrat, Republican, and Independent/Others. Then, if a respondent chose "Democrat" or "Republican", the respondent would see a follow-up question about whether they considered themselves a strong party member or not. If a respondent chose "Independent", they would see a follow-up question about their leaning status, with three options: "Lean toward the Democratic Party," "Lean toward the Republican Party," or "Don't lean either way."

There were two ways of coding this:

For the moderation analysis to test our hypothesis, a dummy variable "Republican" would be created where Republican respondents would be coded as 1, and Democrat and Independent respondents as 0.

A numerical variable on "partisanship" would be created with the following coding: "Strong Republican" as 1, "Not Strong Republican" as 5/6, "Lean toward the Republican Party" as 2/3, "Don't lean either way" as 1/2, "Lean toward the Democratic Party" as 1/3, "Not Strong Democrat" as 1/6, and "Strong Democrat" as 0.

Medical AI May 2024

Start of Block: Consent

In this survey, we will ask your opinions on a variety of topics. The purpose of this research project is to study the opinions of Americans. The survey takes no more than 5 minutes. During this survey, please do not push the "Back" button on your web browser. After you answer a question, you cannot go back to an earlier question to read or change your answer. If you accidentally push the "Back" button, please push the "Refresh" button on your browser so you can continue with the survey. Your participation is voluntary, and your responses are confidential. There are no risks to your participation, and we cannot and do not guarantee or promise that you will receive any benefits from this study other than the financial compensation that has been promised to you. If you have questions about the research project, you may contact Catherine Chen at Stanford University by email at tche101@stanford.edu. If you are not satisfied with how this study is being conducted, or if you have any concerns, complaints, or general questions about the research or your rights as a participant, please contact the Stanford Institutional Review Board (IRB) to speak to someone independent of the research team at (650)-723-2480 or toll free at 1-866-680-2906. You can also email irbnonmed@stanford.edu or write to the Stanford IRB, Stanford University, 1705 El Camino Real, Palo Alto, CA 94306. Please print or save a copy of this page for your records. Please click "Continue" to begin the survey.”

- Continue

End of Block: Consent

Start of Block: Does not mention AI

You're having some moderate symptoms and are thinking about getting advice from a doctor.

Here is Doctor M, a doctor with average experience and expertise in treating your illness.

End of Block: Does not mention AI

Start of Block: No use

You're having some moderate symptoms and are thinking about getting advice from a doctor.

Here is Doctor M, a doctor with average experience and expertise in treating your illness.

Doctor M doesn't use artificial intelligence (AI, such as ChatGPT) at all when evaluating symptoms and deciding on treatments.

End of Block: No use

Start of Block: Moderate use

You're having some moderate symptoms and are thinking about getting advice from a doctor.

Here is Doctor M, a doctor with average experience and expertise in treating your illness.

Doctor M uses artificial intelligence (AI, such as ChatGPT) to a moderate extent when evaluating symptoms and deciding on treatments.

End of Block: Moderate use

Start of Block: Extensive use

You're having some moderate symptoms and are thinking about getting advice from a doctor.

Here is Doctor M, a doctor with average experience and expertise in treating your illness.

Doctor M uses artificial intelligence (AI, such as ChatGPT) extensively when evaluating symptoms and deciding on treatments.

End of Block: Extensive use

Start of Block: DVs

How likely are you to seek medical help from Doctor M?

- Extremely likely
- Very likely
- Moderately likely
- Slightly likely
- Not likely at all

| Page Break |  |
| --- | --- |

To what extent do you trust Doctor M as a person?

- A great deal
- A lot
- A moderate amount
- A little
- None at all

| Page Break |  |
| --- | --- |

To what extent do you trust Doctor M as a professional?

- A great deal
- A lot
- A moderate amount
- A little
- None at all

End of Block: DVs

Start of Block: AI experience

How much do you know about AI, such as ChatGPT?

- A great deal
- A lot
- A moderate amount
- A little
- None at all

Timing

First Click

Last Click

Page Submit

Click Count

| Page Break |  |
| --- | --- |

Over the last six months, how frequently do you use AI, such as ChatGPT?

- Extremely frequently
- Very frequently
- Moderately frequently
- Slightly frequently
- Not frequently at all

Timing

First Click

Last Click

Page Submit

Click Count

End of Block: AI experience

Start of Block: Demographics

What's your biological sex?

- Male
- Female

Timing

First Click

Last Click

Page Submit

Click Count

| Page Break |  |
| --- | --- |

| 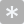 |
| --- |

In what year were you born?

________________________________________________________________

Timing

First Click

Last Click

Page Submit

Click Count

| Page Break |  |
| --- | --- |

What is the highest level of school you have completed or the highest degree you have received?

- Less than 1st grade
- 1st, 2nd, 3rd or 4th grade
- 5th or 6th grade
- 7th or 8th grade
- 9th grade
- 10th grade
- 11th grade
- High school diploma or equivalent (ex. GED)
- Some college but no degree
- Associate degree in college - Occupational/vocational program
- Associate degree in college - Academic program
- Bachelor's degree (ex. BA, AB, BS)
- Master's degree (ex. MA, MS, MEng, MEd, MSW, MBA)
- Professional school degree (ex. MD, DDS, DVM, LLB, JD)
- Doctorate degree (ex. PhD, EdD)

Timing

First Click

Last Click

Page Submit

Click Count

| Page Break |  |
| --- | --- |

The next question is about the total income in 2023 for you and all members of your family who lived with you during 2023, before taxes. Please include money you and all members of your family received from jobs, pensions, social security, interest, dividends, capital gains, profits from businesses, unemployment payments, and all other money received. Adding up the income from all these sources and all other sources, was the total income of you and all members of your family who lived with you in 2023, before taxes, less than $50,000 or was it $50,000 or more?

- Less than $50,000
- $50,000 or more

Timing

First Click

Last Click

Page Submit

Click Count

| Page Break |  |
| --- | --- |

And in which of the following groups was the total income of you and all members of your family who lived with you in 2023, before taxes?

- Less than $10,000
- $10,000 to less than $20,000
- $20,000 to less than $30,000
- $30,000 to less than $40,000
- $40,000 to less than $50,000

Timing

First Click

Last Click

Page Submit

Click Count

| Page Break |  |
| --- | --- |

And in which of the following groups was the total income of you and all members of your family who lived with you in 2023, before taxes?

- $50,000 to less than $75,000
- $75,000 to less than $100,000
- $100,000 to less than $150,000
- $150,000 or more

Timing

First Click

Last Click

Page Submit

Click Count

| Page Break |  |
| --- | --- |

Are you Spanish, Hispanic, or Latino?

- Yes
- No

Timing

First Click

Last Click

Page Submit

Click Count

| Page Break |  |
| --- | --- |

Below please choose one or more races that you consider yourself to be:

- White
- Black or African American
- American Indian or Alaska Native
- Asian
- Native Hawaiian or other Pacific Islander
- Some other race

Timing

First Click

Last Click

Page Submit

Click Count

| Page Break |  |
| --- | --- |

End of Block: Demographics

Start of Block: PID_DR

Do you consider yourself a Democrat, a Republican, an Independent, or what?

- Democrat
- Republican
- Independent
- Other

Timing

First Click

Last Click

Page Submit

Click Count

End of Block: PID_DR

Start of Block: PID_RD

Do you consider yourself a Republican, a Democrat, an Independent, or what?

- Republican
- Democrat
- Independent
- Other

Timing

First Click

Last Click

Page Submit

Click Count

End of Block: PID_RD

Start of Block: PID Strength

Do you lean toward the Democratic Party, lean toward the Republican Party, or do you not lean either way?

- Lean toward the Democratic Party
- Lean toward the Republican Party
- Don't lean either way

Timing

First Click

Last Click

Page Submit

Click Count

| Page Break |  |
| --- | --- |

Do you think of yourself as a strong Republican or a not strong Republican?

- Strong Republican
- Not strong Republican

Timing

First Click

Last Click

Page Submit

Click Count

| Page Break |  |
| --- | --- |

Do you think of yourself as a strong Democrat or a not strong Democrat?

- Strong Democrat
- Not strong Democrat

Timing

First Click

Last Click

Page Submit

Click Count

End of Block: PID Strength

Start of Block: End

Thanks for your participation. Your code is C1D752U3. **Please proceed to the next screen to make sure your answer is properly recorded.**

End of Block: End
